# Medication Errors in a Swiss Cardiovascular Surgery Department: A Cross-Sectional Study Based on a Novel Medication Error Report Method

**DOI:** 10.1155/2013/671820

**Published:** 2013-01-31

**Authors:** Kaspar Küng, Thierry Carrel, Brigitte Wittwer, Sandra Engberg, Natalie Zimmermann, René Schwendimann

**Affiliations:** ^1^Institute of Nursing Science, University of Basel, Bernoullistraß 28, 4056 Basel, Switzerland; ^2^Department of Cardiovascular Surgery, University Hospital of Bern, Freiburgstraß 1, 3000 Bern, Switzerland; ^3^Department of Health Promotion and Development, School of Nursing, University of Pittsburgh, 350 Victoria Building, 3500 Victoria Street, Pittsburgh, PA 15261, USA; ^4^Department of Orthopedic Surgery, University Hospital of Bern, Freiburgstraß 1, 3000 Bern, Switzerland

## Abstract

The purpose of this study was (1) to determine frequency and type of medication errors (MEs), (2) to assess the number of MEs prevented by registered nurses, (3) to assess the consequences of ME for patients, and (4) to compare the number of MEs reported by a newly developed medication error self-reporting tool to the number reported by the traditional incident reporting system. We conducted a cross-sectional study on ME in the Cardiovascular Surgery Department of Bern University Hospital in Switzerland. Eligible registered nurses (*n* = 119) involving in the medication process were included. Data on ME were collected using an investigator-developed medication error self reporting tool (MESRT) that asked about the occurrence and characteristics of ME. Registered nurses were instructed to complete a MESRT at the end of each shift even if there was no ME. All MESRTs were completed anonymously. During the one-month study period, a total of 987 MESRTs were returned. Of the 987 completed MESRTs, 288 (29%) indicated that there had been an ME. Registered nurses reported preventing 49 (5%) MEs. Overall, eight (2.8%) MEs had patient consequences. The high response rate suggests that this new method may be a very effective approach to detect, report, and describe ME in hospitals.

## 1. Introduction

Adverse events (AEs) caused by medication errors (MEs) continue to be one of the great challenges in acute care settings. Recent data suggest that each year more than 1.5 million patients are harmed by ME in the United States [1]. A substantial body of evidence confirms the risk resulting from ME [2–6]. According to the report “Preventing Medication Errors,” ME affect approximately 5% to 10% of patients in the United States and cause more than 7000 deaths annually [1]. 

The definition of ME remains inconsistent although attempts to develop an international definition have been made [7]. The National Coordinating Council for Medication Error Reporting and Prevention (NCC MERP) states that *“A medication error is any preventable event that may cause or lead to inappropriate medication use or patient harm while the medication is in the control of the health care professional, patient, or consumer. Such events may be related to professional practice, health care products, procedures, and systems, including prescribing; order communication; product labeling, packaging, and nomenclature; compounding; dispensing; distribution; administration; education; monitoring; use” [8].*

Studies conducted in various healthcare settings report medication error rates between 19–70%, depending on research methodology [7, 9–13]. High rates continue to exist despite increased levels of awareness about ME in healthcare over the past decade and new developments in technology designed to reduce such errors [14]. One large study analysing when in the medication process ME occurred found that 39% of errors occurred during ordering, 38% during medication administration, 12% during order transcription, and 11% during medication dispensing [15]. Barker and colleagues reported that every fifth medication dose administered resulted in an error, including the wrong time of administration (43%), omission of a dose (30%), wrong dosage (17%), and other errors (10%) [16]. 

Cardiovascular medications have been cited as one of the most common classes of drugs associated with ME [2, 4, 15, 17]. LaPointe and Jollis detected a medication error prevalence rate of 32% in cardiovascular patients. In more than 40% of those MEs, cardiovascular medications were involved [17]. The Adverse Drug Event Prevention Study Group reported 2.4 odds of severe adverse drug events from cardiovascular medications compared to other medications [2, 4, 15]. Hence, ME in cardiovascular settings need special attention, and it can be assumed that cardiovascular patients are at an increased risk for negative consequences from ME. However, the exact rate of ME is difficult to determine due to varying methods of error reporting and calculating error rates. These differences make it difficult to compare data from different studies of ME. The information used to decide whether errors occurred depends strongly on the way in which the information is gathered, and many different methods have been used [18–21]. Examples of methods utilized to collect data on medication errors include critical incidence reporting systems (CIRSs), generally anonymous self-reporting tools used for ME reporting and analysis [22]; incidence reports which typically involve health care providers actively recording information on events [23]; direct observation; trigger tools; chart review; surveys of from health professionals or patients regarding drug related events. These methods can be complementary, and a combination may be useful [19, 20]. However, currently used methods like incident reports or chart reviews are known to underestimate the incidence of ME. Furthermore, most existing tools are not specifically designed to measure ME and may therefore miss certain types of ME. Since no data about pragmatic, easy to use, and low price ME reporting tools exist, new ME reporting approaches—focussing just on ME reporting—are ardently needed.

After an exhausting literature search, no studies utilizing a specific ME self reporting tool—aiming to report ME anonymously—could be identified. To close this gap, we conducted the first ME reporting study according to a novel method in a Swiss cardiovascular surgery department. 

The purpose of this study was to examine ME events in a cardiovascular surgery department of a tertiary level hospital using a new medication error reporting method. 

The specific aims of the study were (1) to determine frequency and type of ME, (2) to assess the number of MEs prevented by registered nurses, (3) to assess the consequences of ME for patients, and (4) to compare the number of MEs reported by a newly developed medication error self reporting tool to the number reported by the traditional incident reporting system.

## 2. Methods

### 2.1. Design

A Cross-sectional study design was used to assess medication error events.

### 2.2. Setting and Sample

The study was conducted in the Cardiovascular Surgery Department of the University Hospital of Bern, Switzerland. The department consists of five units including 60 patient beds. A convenience sample of registered nurses (RNs) involved in direct patient care was recruited. All RNs (*n* = 119) working on the units during the data collection period were eligible to participate in the study. The mean age of the RNs was 36 years (SD 10.8). The mean work experience was 9.5 years (SD 9.0), and the mean work experience in the cardiovascular surgery setting was 5.6 years (SD 5.0). 

### 2.3. Variables

The outcome variables of interest in this study were (1) ME frequency and type, (2) ME prevention by RNs, and (3) ME consequences for patients. 

### 2.4. Unit of Analysis

To calculate the incidence of ME during the one-month study period the unit of analysis in this study was the number of administered medication doses. The amount of administered doses during the data collection period was calculated retrospectively by medical chart review.

### 2.5. Medication Process and Definition

According to Aspden [1], the medication process is a five-step process that encompasses ordering, transcribing, preparing, administering, and monitoring. The clinical medication use process in the study department is in line with this five-step process, and the RNs in our department are responsible in all steps but the ordering. 

After a physician ordered a medication regime for a patient, nurses proofread the prescription, transcribe the handwritten medication orders into a separate medical chart, prepare the medication, according to the written information, administer the medication and monitor the effects after the patient receives the medication. 

In our study, we have considered MEs as errors that occurred during ordering, transcription of the order, and during medication administration. Categories of medication errors were further defined as follows:

1. wrong order: any order that was illegible, incomplete (missing of dosage, medication name, and intended administration method), or wrong (wrong prescription in terms of medication name, dosage, or administration method). If an RN detects an ordering error, the nurse needs to clarify the order with the physician who was involved;
2. wrong transcription: any medication order that was transcribed incorrectly from the physician's ordering sheet to a patient's medical chart by an RN;
3. wrong administration: any medication administration that deviates from at least one of the five rights (right patient, right medication, right dose, right route, and right time).

### 2.6. Measurement

To report ME events, we developed a specific medication error self reporting tool (MESRT) according the study purposes. This tool was composed of 13 items that collected specific data on ME frequency and type (such as errors during the ordering, transcribing, preparing, administering, and monitoring phases), ME prevented by RNs, and patient consequences attributable to ME events (Table 1).

This MESRT is a unique reporting method focussing specifically on ME events rather than addressing a variety of adverse events which might occur in hospitals. The MESRT consists of items to capture errors in the medication use process only. Moreover, we developed the MESRT as a pocket size booklet with 50 sheets (with instructions to complete one for ME event or if there were no errors to complete one at the end of the shift) each. Every registered nurse received a booklet and was instructed to report all MEs after they occurred. Finally, the MESRT allowed nurses to report MEs anonymously. 

### 2.7. Content Validity Testing

Prior to the study, the MESRT was pilot tested with a focus group of 10 randomly selected RNs from the participating cardiovascular surgery units to evaluate its content validity. We calculated the content validity index (CVI), a method used to quantify content validity of multi-item scales [24]. 

A CVI can be computed for each item (I-CVI) on a given scale as well as for the entire scale (S-CVI). To calculate the I-CVI, all participating RNs were asked to rate the relevance of each MESRT item, using a 4-point likert scale (1 = not relevant, 2 = somewhat relevant, 3 = quite relevant, and 4 = highly relevant). Then, for each item, the I-CVI was computed as the number of nurses giving a rating of either 3 or 4, divided by the number of nurses rating the item. The average S-CVI was computed by adding all I-CVIs and dividing this sum by the total number of items (*n* = 13). An average S-CVI of 0.90 or higher is considered to indicate excellent content validity [24]. Our pretest resulted in an average S-CVI of 0.93, reflecting an excellent content validity.

### 2.8. Data Collection Method

RNs were informed about the purpose of the study and how to use the MESRT to report ME events. The principle investigator reviewed the 13 items included in the MESRT and how to respond to them with each of the participating RNs to minimize reporting bias. Nurses were informed by the principle investigator during information rounds one week before the data collection phase. During the data collection period, information rounds were offered daily.

Prior to data collection, each RN received a standardized pocket size booklet with 50 MESRT reporting sheets. RNs were instructed to complete MESRT after each medication error and, if there were no errors during their shift, to complete one indicating that there had been no errors at the end of their shift. Consequently, each RN completed a MESRT at the end of their shift regardless of whether an ME occurred or not. If more than one ME event occurred during the shift, the RN completed a MESRT for each event. Completed MESRTs were dropped in the study-box on every unit. No personal identifying data were collected. Data collection took place from October 1st to 31st of 2009. The MESRT was used in addition to the hospital's critical incident reporting system (CIRS) which is used to voluntarily report AEs including those in the cardiovascular department. In the cardiovascular surgery department, CIRS was implemented in 2006. All RNs were informed that their CIRS reporting practice was not to be interrupted during the one-month study period and that all AEs should be reported through CIRS as usual, independently of completing the MESRT. 

### 2.9. Data Analysis

Collected MESRT data were entered into SPSS for Windows (V16.0, SPSS, Inc., Chicago, IL) by the principle investigator. To determine ME frequency and type, number of MEs prevented by RNs, and patient consequences attributable to ME, data were analyzed using descriptive statistic methods (frequencies, percentages, means, and standard deviations). 

### 2.10. Ethical Considerations

As voluntary reporting and disclosure of ME may pose a highly sensitive issue among health care professionals, identity protection and confidential use of data were guaranteed. Participants provided written informed consent prior to the data collection, and self-reporting was voluntary during data collection. Our study was approved by the Ethical Board of the Canton of Bern.

## 3. Results

During the one-month study period, a total of 24, 617 medication doses were administered to cardiovascular patients. During this timeframe, 288 MEs were reported on MESRTs. The incidence of ME in this study was 1.2% based on reported ME in relation to the total administered medication doses. 

A total of 987 MESRTs were returned (response rate 84%). In fact, 650 (66%) RNs reported that no ME occurred during their shift. A total of 288 of the MESRTs (29%) reported an ME. RNs reported preventing 49 MEs (5% of completed MESRTs). Of the 288 reported MEs, eight (2.8%) revealed information that ME had a consequence for a patient such as extra patient monitoring and increased surveillance frequency. However, no patient experienced harmful or life threatening ME consequences during the study period. 

The most frequently reported type of ME in our study was administering the medication at the wrong time (48.3%). MEs were categorized as errors that occurred when the medication was ordered, during transcription of the order, and during medication administration (Table 2). Twenty-nine percent of MEs occurred during ordering, 13% during transcription, and 58% during medication administration. 

Comparatively, during the study period, seven ME events were reported in the CIRS of the cardiovascular surgery department. The reported CIRS cases were transcription and administration errors and were categorized as follows: transcription errors (*n* = 2), wrong dose (*n* = 3), wrong time (*n* = 1), and wrong medication (*n* = 1).

## 4. Discussion

In this study, we successfully implemented a novel reporting method—a medication error self reporting tool (MESRT)—to examine the frequency and type of ME, their consequences for patients and the number of MEs prevented by RNs.

The overall ME incidence in our study was 1.2%. ME incidence was calculated based on number of reported MEs relative to the total number of medication doses administered during the study period. If the patient had been the unit of analysis, ME frequency would probably be significantly higher. Hence, the methodological approach must be considered when interpreting these findings [18]. Most MEs reported with the MESRT were medication administration errors (58%) followed by ordering errors (29%) and transcription errors (13%). Our results on ME types are similar to results of Leape and colleagues who indicated that 49% of MEs occurred during administration of medications, 39% occurred during physician ordering, and 12% during the transcription phase [15]. Bates and colleagues reported that 56% of MEs occurred during the ordering stage, 6% during transcription, and 34% during medication administration [2]. Our results are in line with these data.

Detected MEs were mainly medication administration errors followed by ordering and transcription errors. All medication orders were handwritten by physicians and transcribed by an RN. Transcription errors accounted for 12% of the reported MEs. One effective strategy to reduce ordering and transcription errors may be the use of a computerized physician ordering entry system combined with barcode medication administration record [3, 25–28]. 

Among all medication administration errors, time errors were the most prevalent. For the purpose of this study, a time error was defined as administering a medication more than 30 minutes before or after its scheduled administration time. Hence, the high rate of time errors may be related to the timeframe used. Unfortunately, there is no broad agreement in the literature regarding what constitutes early or late medication administration [16, 28, 29].

According to all analyzed MESRTs, 5% of potential ME were prevented by RNs. This is surprising since RNs could be seen as last barriers before a medication reach a patient. However, the reason for this relatively low rate may be the self reporting method utilized in this study. 

No ME reported in our study had a negative impact on patients' health. No patient was harmed by a medication. Hence, no patients experienced an adverse drug event (ADE), although cardiovascular patient are at high risk for negative consequences from ME. In contrast, Bates et al. reported an ADE rate of 6.5%, with 7% of ME judged to be associated with potential ADEs [2]. Similarly, Hardmeier and colleagues reported that 7.5% of patients experienced an ME-associated ADE during hospitalisation [30]. 

During the timeframe of this study, many more MEs were reported with the MESRT (*n* = 288) than with the traditional CIRS (*n* = 7). Our findings are similar to the findings of other studies and support the argument that reporting attitudes may vary greatly, depending on the ME assessment methods used [7, 18, 20, 31, 32]. Moreover, the findings suggest that CIRS may not reliably measure the prevalence of ME. However, various other factors such as disparities in ME definition, terminology, and unequal reporting tools may influence ME reporting [7, 13, 19, 33]. 

The remarkable disparity between the MEs captured with the MESRT and those reported by CIRS might be a crucial finding concerning error reporting accuracy and should be considered when designing ME reporting methods in the future. Our data show that it is important to use simple reporting tools which facilitate ME reporting. Clearly defined ME reporting standards and measurement tools are needed to facilitate ME reporting and to ensure data accuracy [15, 31, 34]. 

The findings of this study suggest that our MESRT may facilitate ME reporting substantially. The MESRT we developed is easy to use, and it is clear for RNs what to report as an ME. No login into a computer system is required to complete a MESRT. Some RNs may have been concerned that by requiring computer login to report an ME through CIRS, reporting was not truly anonymous. The MESRT used in this study respects anonymous reporting rigorously. The standardized pocket size booklet allows an RN to anonymously report an ME right after its occurrence. By reporting the ME anonymously, fear of blame as a result of reporting an error can be avoided. This is pivotal because one of the overriding barriers to report adverse events is fear of blame and punishment, depending on the existing safety culture in an institution [35–39]. 

## 5. Limitations

Although this was the first study to systematically report ME in a Swiss cardiovascular hospital setting with a new ME reporting method, potential limitations have to be considered. First, the study was conducted over a short period of time which may not have been representative of usual medication error events. Second, the reporting of ME with a customized MESRT may represent some degree of underreporting due to the fact that not all MEs are consciously detected by RNs. In addition, medication omission errors and administering unauthorized drugs were not captured by the MESRT. Finally, the results must be carefully interpreted. All of the nurses worked in one cardiovascular surgery department. Findings may, therefore, not be generalized to other units and hospitals. However, our study does provide a snapshot of ME within a given period of time and suggests that rates are probably much higher than those detected by CIRS. This study should be replicated within a larger sample and over a longer period of time in acute care settings. Despite its limitations, this study demonstrated the successful use of a novel ME reporting method that could provide insights into the occurrence of ME and provide a basis for reviewing medication safety procedures. 

## 6. Conclusions

The results of this study are a novelty for Switzerland since data on ME are sparsely available in Switzerland, and data from cardiovascular departments are—to our knowledge—nonexistent so far. Therefore, this is the first Swiss study that utilized a specific MESRT to collect ME data. 

During a one-month data collection period, 987 MESRTs were completed. Overall, 288 MEs were reported by RNs. Relative to the total number of doses on medication administered during the time period of this study, the incidence of ME was 1.2%. 

Surprisingly, our novel approach had the potential to detect significantly more MEs than the hospital CIRS. The high response rate suggests that this new ME reporting tool, for at least short-term monitoring, may be a very effective approach to detect, report, and describe ME. The MESRT we developed is a simple tool for RNs that appears to be an acceptable method of reporting medication error events. It detected many more MEs than usual incidence reporting systems. Future studies should examine its utility in a larger sample of nurses conducted over a longer period of time in multiple hospitals and in combination with other medication assessment methods such as direct observation. With respect to further studies, it will be of great value to expand the MESRT with more items asking about, for example, omission errors and administration of unauthorized drugs.

## Figures and Tables

**Table 1:** The medication error self reporting tool** (**MESRT) to report medication error events.

During my shift, one of the following medication error-related events occurred (please mark with a cross)	
(1) □ I administered a *wrong medication* to a patient	
(2) □ I administered a *medication* at the *wrong time* (more than 30 minutes earlier or later than ordered)	
(3) □ I administered a *medication in a wrong dosage *	
(4) □ I administered a *medication to the wrong patient *	
(5) □ I administered a *medication the wrong route *	

(6) □ A medication prescription was *illegible *	
(7) □ A medication prescription was *incomplete *	
(8) □ A medication prescription was *wrong *	
(9) □ A medication prescription was *transcribed wrong *	

(10) □ The medication error event *had no consequences* for the patient	
(11) □ The medication error event *had consequences* for the patient	
→ If yes, what consequences? (use the space below)	
	
	

(12) □ I realised that there is an error involved, but I was able to prevent the error before it happened or resulted in patient harm	
→ If yes, what kind of error could be prevented? (use the space below)	
	
	

(13) □ No medication error-related event happened to me during my shift	

□ Morning shift □ Evening shift □ Night shift Date:	

**Table 2:** Types, frequencies, and categories of medication errors.

Type of medication error	*N* (%)	Category
Wrong time	139 (48.3)	C
Wrong transcription	38 (13.2)	B
Incomplete prescription	33 (11.5)	A
Illegible prescription	32 (11.0)	A
Wrong dose	20 (7.0)	C
Wrong prescription	19 (6.6)	A
Wrong medication	6 (2.1)	C
Wrong route	1 (0.4)	C
Wrong patient	0 (0)	C

A: ordering error; B: transcribing error; C: administration error.
